# 4-(Naphthalene-2-carboxamido)­pyridin-1-ium thio­cyanate–*N*-(pyridin-4-yl)naphthalene-2-carboxamide (1/1)

**DOI:** 10.1107/S1600536812039864

**Published:** 2012-09-26

**Authors:** Sohail Saeed, Naghmana Rashid, Ray J. Butcher, Sema Öztürk Yildirim, Rizwan Hussain

**Affiliations:** a Department of Chemistry, Research Complex, Allama Iqbal Open University, Islamabad 44000, Pakistan; b Department of Chemistry, Howard University, 525 College Street NW, Washington, DC 20059, USA; c Department of Physics, Faculty of Sciences, Erciyes University, 38039 Kayseri, Turkey; d National Engineering & Scientific Commission, PO Box 2801, Islamabad, Pakistan

## Abstract

The asymmetric unit of the title compound, C_16_H_13_N_2_O^+^·NCS^−^·C_16_H_12_N_2_O, contains two *N*-(pyridin-4-yl)naphthalene-2-carboxamide mol­ecules, both are partially protonated in the pyridine moiety, *i.e.* the H atom attached to the pyridine N atom is partially occupied with an occupancy factor of 0.61 (3) and 0.39 (3), respectively. In the crystal, protonated and neutral *N*-(pyridin-4-yl)naphthalene-2-carboxamide mol­ecules are linked by N—H⋯N hydrogen bonding; the thio­cyanate counter-ion links with both protonated and neutral *N*-(pyridin-4-yl)naphthalene-2-carboxamide mol­ecules *via* N—H⋯S and N—H⋯N hydrogen bonding. The dihedral angles between the pyridine ring and naphthalene ring systems are 11.33 (6) and 9.51 (6)°, respectively. π–π stacking is observed in the crystal structure, the shortest centroid–centroid distance being 3.5929 (8) Å. The crystal structure was determined from a nonmerohedral twin {ratio of the twin components = 0.357 (1):0.643 (1) and twin law [-100 0-10 -101]}.

## Related literature

For background to the biological activity of *N*-substituted benzamides and their applications in synthesis, see: Saeed *et al.* (2011 ▶); Priya *et al.* (2005 ▶). For related structures and use in mol­ecular recognition, see: Toda *et al.* (1987 ▶); Saeed *et al.* (2011 ▶, 2012 ▶). For bond lengths, see: Allen *et al.* (1987 ▶).
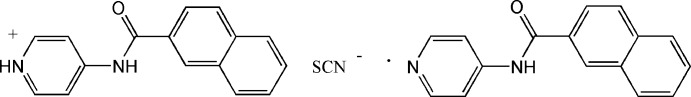


## Experimental

### 

#### Crystal data

C_16_H_13_N_2_O^+^·NCS^−^·C_16_H_12_N_2_O
*M* *_r_* = 555.64
Triclinic, 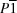

*a* = 8.2667 (4) Å
*b* = 12.9008 (7) Å
*c* = 13.5579 (8) Å
α = 84.047 (5)°
β = 77.566 (5)°
γ = 71.684 (5)°
*V* = 1339.45 (13) Å^3^
*Z* = 2
Cu *K*α radiation
μ = 1.41 mm^−1^
*T* = 123 K
0.38 × 0.35 × 0.29 mm

#### Data collection

Xcalibur (Ruby, Gemini) diffractometer
Absorption correction: multi-scan (*CrysAlis RED*; Agilent, 2011 ▶)*T*_min_ = 0.904, *T*_max_ = 1.000
9408 measured reflections
9408 independent reflections
8399 reflections with *I* > 2σ(*I*)

#### Refinement

*R*[*F* ^2^ > 2σ(*F* ^2^)] = 0.047
*wR*(*F* ^2^) = 0.137
*S* = 1.02
9408 reflections
374 parameters
H-atom parameters constrained
Δρ_max_ = 0.36 e Å^−3^
Δρ_min_ = −0.30 e Å^−3^

### 

Data collection: *CrysAlis PRO* (Agilent, 2011 ▶); cell refinement: *CrysAlis PRO*; data reduction: *CrysAlis PRO*; program(s) used to solve structure: *SHELXS97* (Sheldrick, 2008 ▶); program(s) used to refine structure: *SHELXL97* (Sheldrick, 2008 ▶); molecular graphics: *SHELXTL* (Sheldrick, 2008 ▶); software used to prepare material for publication: *SHELXTL*.

## Supplementary Material

Crystal structure: contains datablock(s) I, global. DOI: 10.1107/S1600536812039864/xu5617sup1.cif

Structure factors: contains datablock(s) I. DOI: 10.1107/S1600536812039864/xu5617Isup2.hkl

Supplementary material file. DOI: 10.1107/S1600536812039864/xu5617Isup3.cml

Additional supplementary materials:  crystallographic information; 3D view; checkCIF report

## Figures and Tables

**Table 1:** Hydrogen-bond geometry (Å, °)

*D*—H⋯*A*	*D*—H	H⋯*A*	*D*⋯*A*	*D*—H⋯*A*
N1*A*—H1*AA*⋯N1*S*	0.88	2.24	3.0576 (17)	154
N1*B*—H1*BA*⋯S1*S* ^i^	0.88	2.69	3.5345 (11)	162
N2*A*—H2*AA*⋯N2*B*	0.88	1.77	2.6492 (15)	173
N2*B*—H2*BB*⋯N2*A*	0.88	1.77	2.6492 (15)	177

Symmetry code: (i) 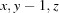.

**Fig. 1.**
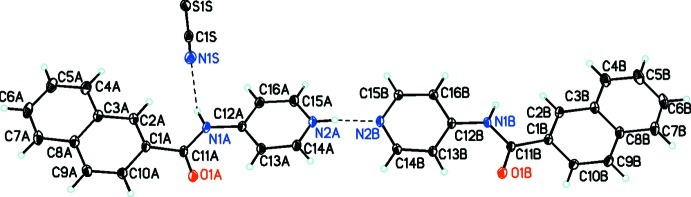
The molecular structure of the title compound with labeling and displacement ellipsoids drawn at the 30% probability level. The dashed line denotes the hydrogen bond between the neutral and protonated moieties.

**Fig. 2.**
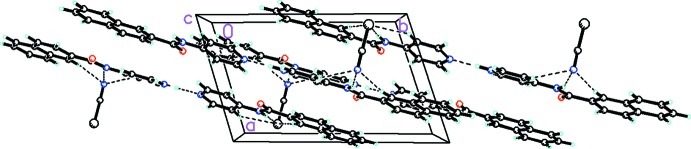
The molecular packing of the title compound, viewed down c axis. The dashed lines denote hydrogen bonds.

## supplementary crystallographic information

### Comment

Amides are known to play a pivital role in molecular recognition, being important components in supramolecular chemical anion sensors technology (Saeed *et al.*, 2011, 2012). Moreover, amides have also been reported as antimicrobial agents (Priya *et al.*, 2005). A compound with the same basic skeleton as the title compound has been used in host–guest chemistry to form numerous highly crystalline adducts with a variety of common organic solvents (Toda *et al.*, 1987). The structure of the title compound has been determined to explore the effect of substituents on the structure of the title compound.

The crystal structure of the title compound (Fig. 1), [(C_16_H_13_N_2_O^+^)(NCS^-^)] C_16_H_12_N_2_O, there are two independent molecules (A and B) in the asymmetric unit of which one is protonated (A). In both A and B the naphthyl and pyridine rings are planar with a mean deviation from the least- squares plane defined by naphthyl ring carbon atoms (C1—C10) and C1A of -0.018 (1) Å and 0.0268 (1) Å for the C1B. All bond lengths and angles in (I) are within normal ranges (Allen *et al.*, 1987). The dihedral angles between the naphthyl and pyridine ring systems is 11.33 (6) and 9.51 (6)° in molecules A and B, respectively. In the crystal, N—H···N and N—H···S intra- and intermolecular hydrogen bonds are observed (Table 1) as well as weak π - π stacking interactions [*Cg*1···*Cg*4 (*x*,1 + *y*,*z*] = 3.615 (1) Å, *Cg*2···*Cg*3 (*x*,-1 + *y*,*z*] = 3.593 (1) Å, *Cg*2···*Cg*5 (*x*,-1 + *y*,*z*] = 3.735 (1) Å and *Cg*3···*Cg*4 (1 + *x*,1 + *y*,*y*] = 3.686 (1) Å where *Cg*1(N2A/C12A—C16A), *Cg*2(N2B/C12B—C16B), *Cg*3(C1A—C3A/C8A—C10A), *Cg*4(C1B—C3B/C8B—C10B) and *Cg*5(C3A—C8A) are the centroids of the pyridinium and naphthalene rings](Fig. 2).

### Experimental

A solution of naphthalene 2-carbonyl chloride (0.01 mol) in anhydrous acetone (80 ml) was added dropwise to a suspension of dry sodium thiocyanate (0.01 mol) in acetone (50 ml) and the reaction mixture was refluxed for 45 min. After cooling to room temperature, a solution of 4-aminopyridine (0.01 mol) in anhydrous acetone (25 ml) was added and the resulting mixture refluxed for 2 h. Hydrochloric acid (0.1 N, 400 ml) was added, and the solution was filtered.

### Refinement

The crystal structure was solved from non-merohedral twin with the twin law in the reciprocal matrix of -1, 0, 0: 0, -1, 0: -1, 0, 1 and the twin component ratio of 0.357 (1)/0.643 (1). In the refinement the HKLF 5 reflection file format in *SHELXL* was used.

H atoms attached to C atoms were positioned with idealized geometry and were refined isotropic with *U*_eq_(H) set to 1.2 times of the *U*_eq_(C) using a riding model with C—H = 0.95 Å. H atoms attached to N atoms were positioned with idealized geometry and were refined isotropically with *U*_eq_(H) set to 1.2 times of *U*_eq_(N) using a riding model with N—H = 0.88 Å. The H atom attached to the pyridine-N atom is disordered over two sites in a ratio of 0.61 (3):0.39 (3).

### Crystal data

**Table:**

C_16_H_13_N_2_O^+^·NCS^−^·C_16_H_12_N_2_O	*Z* = 2
*M**_r_* = 555.64	*F*(000) = 580
Triclinic, *P*1	*D*_x_ = 1.378 Mg m^−^^3^
Hall symbol: -P 1	Cu *K*α radiation, λ = 1.54178 Å
*a* = 8.2667 (4) Å	Cell parameters from 3931 reflections
*b* = 12.9008 (7) Å	θ = 3.3–75.5°
*c* = 13.5579 (8) Å	µ = 1.41 mm^−^^1^
α = 84.047 (5)°	*T* = 123 K
β = 77.566 (5)°	Prism, colorless
γ = 71.684 (5)°	0.38 × 0.35 × 0.29 mm
*V* = 1339.45 (13) Å^3^	

### Data collection

**Table:**

Xcalibur (Ruby, Gemini) diffractometer	9408 independent reflections
Radiation source: Enhance (Cu) X-ray Source	8399 reflections with *I* > 2σ(*I*)
Graphite monochromator	*R*_int_ = 0.000
Detector resolution: 10.5081 pixels mm^-1^	θ_max_ = 76.0°, θ_min_ = 3.3°
ω scans	*h* = −10→10
Absorption correction: multi-scan [*CrysAlis RED* (Agilent, 2011) based on expressions derived from Clark & Reid (1995)]	*k* = −16→16
*T*_min_ = 0.904, *T*_max_ = 1.000	*l* = −12→16
9408 measured reflections	

### Refinement

**Table:**

Refinement on *F*^2^	Primary atom site location: structure-invariant direct methods
Least-squares matrix: full	Secondary atom site location: difference Fourier map
*R*[*F*^2^ > 2σ(*F*^2^)] = 0.047	Hydrogen site location: inferred from neighbouring sites
*wR*(*F*^2^) = 0.137	H-atom parameters constrained
*S* = 1.02	*w* = 1/[σ^2^(*F*_o_^2^) + (0.0899*P*)^2^ + 0.2899*P*] where *P* = (*F*_o_^2^ + 2*F*_c_^2^)/3
9408 reflections	(Δ/σ)_max_ = 0.001
374 parameters	Δρ_max_ = 0.36 e Å^−^^3^
0 restraints	Δρ_min_ = −0.30 e Å^−^^3^

### Special details

**Table:**

Experimental. CrysAlis RED, (Agilent, 2011) Empirical absorption correction using spherical harmonics, implemented in SCALE3 ABSPACK scaling algorithm (Clark & Reid, 1995).
Geometry. All e.s.d.'s (except the e.s.d. in the dihedral angle between two l.s. planes) are estimated using the full covariance matrix. The cell e.s.d.'s are taken into account individually in the estimation of e.s.d.'s in distances, angles and torsion angles; correlations between e.s.d.'s in cell parameters are only used when they are defined by crystal symmetry. An approximate (isotropic) treatment of cell e.s.d.'s is used for estimating e.s.d.'s involving l.s. planes.
Refinement. Refinement of *F*^2^ against ALL reflections. The weighted *R*-factor *wR* and goodness of fit *S* are based on *F*^2^, conventional *R*-factors *R* are based on *F*, with *F* set to zero for negative *F*^2^. The threshold expression of *F*^2^ > σ(*F*^2^) is used only for calculating *R*-factors(gt) *etc*. and is not relevant to the choice of reflections for refinement. *R*-factors based on *F*^2^ are statistically about twice as large as those based on *F*, and *R*- factors based on ALL data will be even larger.

### Fractional atomic coordinates and isotropic or equivalent isotropic displacement parameters (Å^2^)

**Table:**

	*x*	*y*	*z*	*U*_iso_*/*U*_eq_	Occ. (<1)
S1S	0.08493 (5)	0.80651 (3)	0.97576 (3)	0.03396 (11)	
N1S	0.4395 (2)	0.68666 (11)	0.95127 (10)	0.0399 (3)	
C1S	0.2927 (2)	0.73545 (11)	0.96208 (10)	0.0315 (3)	
O1A	0.66272 (14)	0.64980 (8)	0.56439 (7)	0.0319 (2)	
N1A	0.58653 (14)	0.61521 (8)	0.73364 (8)	0.0240 (2)	
H1AA	0.5779	0.6394	0.7935	0.034 (4)*	
N2A	0.44101 (14)	0.33301 (8)	0.74169 (8)	0.0245 (2)	
H2AA	0.4093	0.2735	0.7445	0.029*	0.61 (3)
C1A	0.68983 (17)	0.77349 (10)	0.67251 (10)	0.0245 (3)	
C2A	0.66122 (17)	0.81223 (11)	0.76696 (10)	0.0263 (3)	
H2AB	0.6133	0.7745	0.8239	0.032*	
C3A	0.70214 (18)	0.90814 (11)	0.78109 (11)	0.0276 (3)	
C4A	0.6715 (2)	0.94936 (12)	0.87865 (12)	0.0358 (3)	
H4AA	0.6243	0.9120	0.9362	0.043*	
C5A	0.7098 (2)	1.04289 (13)	0.89019 (14)	0.0412 (4)	
H5AA	0.6893	1.0700	0.9557	0.049*	
C6A	0.7797 (2)	1.09912 (12)	0.80502 (14)	0.0401 (4)	
H6AA	0.8058	1.1638	0.8138	0.048*	
C7A	0.81019 (19)	1.06170 (12)	0.71064 (13)	0.0356 (3)	
H7AA	0.8561	1.1009	0.6541	0.043*	
C8A	0.77348 (17)	0.96363 (11)	0.69583 (11)	0.0277 (3)	
C9A	0.80472 (18)	0.92153 (11)	0.59920 (11)	0.0295 (3)	
H9AA	0.8545	0.9577	0.5416	0.035*	
C10A	0.76400 (17)	0.82912 (10)	0.58763 (10)	0.0268 (3)	
H10A	0.7856	0.8019	0.5221	0.032*	
C11A	0.64576 (16)	0.67468 (10)	0.65080 (10)	0.0239 (3)	
C12A	0.53898 (16)	0.52088 (10)	0.73240 (10)	0.0218 (2)	
C13A	0.53009 (17)	0.47683 (10)	0.64435 (10)	0.0242 (3)	
H13A	0.5583	0.5106	0.5800	0.029*	
C14A	0.47968 (17)	0.38366 (10)	0.65253 (10)	0.0256 (3)	
H14A	0.4721	0.3544	0.5928	0.031*	
C15A	0.45085 (19)	0.37358 (11)	0.82714 (10)	0.0281 (3)	
H15A	0.4240	0.3371	0.8902	0.034*	
C16A	0.49897 (18)	0.46677 (11)	0.82490 (10)	0.0273 (3)	
H16A	0.5050	0.4942	0.8859	0.033*	
O1B	0.27783 (14)	−0.15477 (8)	0.54771 (7)	0.0330 (2)	
N1B	0.21021 (14)	−0.12707 (8)	0.71690 (8)	0.0225 (2)	
H1BA	0.1676	−0.1533	0.7758	0.034 (4)*	
N2B	0.35165 (14)	0.15425 (8)	0.73472 (8)	0.0242 (2)	
H2BB	0.3810	0.2132	0.7397	0.029*	0.39 (3)
C1B	0.16169 (16)	−0.28346 (10)	0.65252 (10)	0.0221 (2)	
C2B	0.10170 (17)	−0.32263 (10)	0.74599 (10)	0.0242 (3)	
H2BA	0.0943	−0.2846	0.8040	0.029*	
C3B	0.05008 (17)	−0.41902 (10)	0.75834 (10)	0.0255 (3)	
C4B	−0.0151 (2)	−0.45925 (13)	0.85460 (12)	0.0357 (3)	
H4BA	−0.0282	−0.4202	0.9129	0.043*	
C5B	−0.0597 (2)	−0.55389 (13)	0.86471 (13)	0.0401 (4)	
H5BA	−0.1037	−0.5800	0.9298	0.048*	
C6B	−0.0404 (2)	−0.61291 (12)	0.77879 (14)	0.0376 (4)	
H6BA	−0.0690	−0.6795	0.7867	0.045*	
C7B	0.01863 (19)	−0.57544 (11)	0.68477 (12)	0.0322 (3)	
H7BA	0.0299	−0.6157	0.6275	0.039*	
C8B	0.06374 (16)	−0.47632 (10)	0.67138 (11)	0.0257 (3)	
C9B	0.12318 (18)	−0.43331 (11)	0.57536 (11)	0.0277 (3)	
H9BA	0.1309	−0.4701	0.5166	0.033*	
C10B	0.16984 (17)	−0.33960 (10)	0.56551 (10)	0.0255 (3)	
H10B	0.2081	−0.3116	0.5001	0.031*	
C11B	0.22166 (16)	−0.18353 (10)	0.63304 (9)	0.0221 (2)	
C12B	0.25886 (16)	−0.03295 (10)	0.71828 (10)	0.0212 (2)	
C13B	0.34762 (17)	0.01249 (10)	0.63456 (10)	0.0239 (3)	
H13B	0.3773	−0.0193	0.5705	0.029*	
C14B	0.39169 (17)	0.10469 (11)	0.64657 (10)	0.0259 (3)	
H14B	0.4536	0.1345	0.5894	0.031*	
C15B	0.26547 (18)	0.11074 (11)	0.81480 (10)	0.0273 (3)	
H15B	0.2363	0.1450	0.8777	0.033*	
C16B	0.21700 (18)	0.01874 (11)	0.81046 (10)	0.0267 (3)	
H16B	0.1560	−0.0094	0.8692	0.032*	

### Atomic displacement parameters (Å^2^)

**Table:**

	*U*^11^	*U*^22^	*U*^33^	*U*^12^	*U*^13^	*U*^23^
S1S	0.0457 (2)	0.03312 (19)	0.02666 (18)	−0.02096 (15)	0.00028 (14)	−0.00364 (13)
N1S	0.0512 (9)	0.0364 (7)	0.0346 (7)	−0.0163 (6)	−0.0070 (6)	−0.0049 (5)
C1S	0.0538 (10)	0.0279 (6)	0.0200 (6)	−0.0233 (7)	−0.0048 (6)	−0.0025 (5)
O1A	0.0463 (6)	0.0275 (5)	0.0268 (5)	−0.0190 (4)	−0.0050 (4)	−0.0023 (4)
N1A	0.0306 (6)	0.0186 (5)	0.0273 (5)	−0.0111 (4)	−0.0076 (4)	−0.0040 (4)
N2A	0.0276 (5)	0.0188 (5)	0.0303 (6)	−0.0107 (4)	−0.0064 (4)	−0.0020 (4)
C1A	0.0235 (6)	0.0179 (6)	0.0335 (7)	−0.0069 (5)	−0.0073 (5)	−0.0014 (5)
C2A	0.0284 (6)	0.0225 (6)	0.0302 (7)	−0.0107 (5)	−0.0066 (5)	0.0005 (5)
C3A	0.0281 (6)	0.0230 (6)	0.0340 (7)	−0.0076 (5)	−0.0105 (5)	−0.0018 (5)
C4A	0.0432 (8)	0.0308 (7)	0.0394 (8)	−0.0149 (6)	−0.0129 (7)	−0.0057 (6)
C5A	0.0468 (9)	0.0340 (8)	0.0512 (10)	−0.0151 (7)	−0.0186 (7)	−0.0111 (7)
C6A	0.0384 (8)	0.0248 (7)	0.0664 (11)	−0.0117 (6)	−0.0223 (7)	−0.0106 (7)
C7A	0.0323 (7)	0.0240 (6)	0.0549 (9)	−0.0119 (6)	−0.0134 (7)	0.0001 (6)
C8A	0.0238 (6)	0.0228 (6)	0.0384 (7)	−0.0070 (5)	−0.0103 (5)	0.0000 (5)
C9A	0.0298 (7)	0.0243 (6)	0.0349 (7)	−0.0106 (5)	−0.0061 (6)	0.0038 (5)
C10A	0.0282 (6)	0.0224 (6)	0.0294 (7)	−0.0078 (5)	−0.0053 (5)	−0.0002 (5)
C11A	0.0233 (6)	0.0163 (5)	0.0332 (7)	−0.0068 (5)	−0.0068 (5)	−0.0010 (5)
C12A	0.0216 (6)	0.0171 (5)	0.0280 (6)	−0.0064 (4)	−0.0059 (5)	−0.0022 (5)
C13A	0.0281 (6)	0.0229 (6)	0.0255 (6)	−0.0119 (5)	−0.0065 (5)	−0.0017 (5)
C14A	0.0303 (6)	0.0230 (6)	0.0276 (6)	−0.0121 (5)	−0.0067 (5)	−0.0038 (5)
C15A	0.0361 (7)	0.0245 (6)	0.0266 (6)	−0.0132 (5)	−0.0073 (5)	0.0010 (5)
C16A	0.0363 (7)	0.0251 (6)	0.0235 (6)	−0.0122 (5)	−0.0063 (5)	−0.0032 (5)
O1B	0.0489 (6)	0.0308 (5)	0.0251 (5)	−0.0232 (4)	−0.0024 (4)	−0.0017 (4)
N1B	0.0290 (5)	0.0188 (5)	0.0229 (5)	−0.0128 (4)	−0.0041 (4)	0.0003 (4)
N2B	0.0276 (5)	0.0176 (5)	0.0303 (6)	−0.0109 (4)	−0.0051 (4)	−0.0017 (4)
C1B	0.0224 (6)	0.0181 (6)	0.0275 (6)	−0.0068 (4)	−0.0072 (5)	−0.0018 (5)
C2B	0.0292 (6)	0.0216 (6)	0.0259 (6)	−0.0110 (5)	−0.0085 (5)	−0.0018 (5)
C3B	0.0277 (6)	0.0217 (6)	0.0308 (7)	−0.0109 (5)	−0.0098 (5)	0.0025 (5)
C4B	0.0457 (8)	0.0344 (7)	0.0343 (8)	−0.0219 (6)	−0.0118 (6)	0.0063 (6)
C5B	0.0481 (9)	0.0370 (8)	0.0442 (9)	−0.0261 (7)	−0.0158 (7)	0.0148 (7)
C6B	0.0366 (8)	0.0232 (6)	0.0605 (10)	−0.0167 (6)	−0.0187 (7)	0.0096 (6)
C7B	0.0310 (7)	0.0212 (6)	0.0495 (8)	−0.0102 (5)	−0.0143 (6)	−0.0027 (6)
C8B	0.0229 (6)	0.0186 (6)	0.0376 (7)	−0.0064 (5)	−0.0098 (5)	−0.0014 (5)
C9B	0.0297 (7)	0.0238 (6)	0.0317 (7)	−0.0083 (5)	−0.0065 (5)	−0.0079 (5)
C10B	0.0288 (6)	0.0232 (6)	0.0258 (6)	−0.0088 (5)	−0.0049 (5)	−0.0040 (5)
C11B	0.0249 (6)	0.0194 (6)	0.0238 (6)	−0.0088 (5)	−0.0052 (5)	−0.0011 (5)
C12B	0.0225 (6)	0.0175 (5)	0.0259 (6)	−0.0077 (4)	−0.0072 (5)	−0.0002 (4)
C13B	0.0290 (6)	0.0223 (6)	0.0234 (6)	−0.0124 (5)	−0.0041 (5)	−0.0014 (5)
C14B	0.0287 (6)	0.0237 (6)	0.0276 (6)	−0.0128 (5)	−0.0040 (5)	0.0007 (5)
C15B	0.0320 (7)	0.0244 (6)	0.0280 (7)	−0.0133 (5)	−0.0015 (5)	−0.0059 (5)
C16B	0.0314 (7)	0.0255 (6)	0.0252 (6)	−0.0145 (5)	−0.0007 (5)	−0.0013 (5)

### Geometric parameters (Å, º)

**Table:**

S1S—C1S	1.6530 (17)	C16A—H16A	0.9500
N1S—C1S	1.163 (2)	O1B—C11B	1.2163 (16)
O1A—C11A	1.2141 (17)	N1B—C11B	1.3836 (16)
N1A—C11A	1.3822 (17)	N1B—C12B	1.3969 (15)
N1A—C12A	1.3941 (15)	N1B—H1BA	0.8800
N1A—H1AA	0.8800	N2B—C15B	1.3392 (17)
N2A—C14A	1.3384 (17)	N2B—C14B	1.3424 (17)
N2A—C15A	1.3476 (17)	N2B—H2BB	0.8800
N2A—H2AA	0.8800	C1B—C2B	1.3642 (18)
C1A—C2A	1.3688 (19)	C1B—C10B	1.4258 (17)
C1A—C10A	1.4201 (19)	C1B—C11B	1.5007 (17)
C1A—C11A	1.5035 (17)	C2B—C3B	1.4199 (17)
C2A—C3A	1.4220 (18)	C2B—H2BA	0.9500
C2A—H2AB	0.9500	C3B—C4B	1.413 (2)
C3A—C8A	1.413 (2)	C3B—C8B	1.4213 (19)
C3A—C4A	1.419 (2)	C4B—C5B	1.368 (2)
C4A—C5A	1.372 (2)	C4B—H4BA	0.9500
C4A—H4AA	0.9500	C5B—C6B	1.411 (2)
C5A—C6A	1.413 (3)	C5B—H5BA	0.9500
C5A—H5AA	0.9500	C6B—C7B	1.360 (2)
C6A—C7A	1.359 (2)	C6B—H6BA	0.9500
C6A—H6AA	0.9500	C7B—C8B	1.4247 (18)
C7A—C8A	1.4330 (19)	C7B—H7BA	0.9500
C7A—H7AA	0.9500	C8B—C9B	1.411 (2)
C8A—C9A	1.411 (2)	C9B—C10B	1.3659 (18)
C9A—C10A	1.3685 (19)	C9B—H9BA	0.9500
C9A—H9AA	0.9500	C10B—H10B	0.9500
C10A—H10A	0.9500	C12B—C13B	1.3943 (17)
C12A—C16A	1.3981 (18)	C12B—C16B	1.4029 (18)
C12A—C13A	1.3993 (17)	C13B—C14B	1.3828 (17)
C13A—C14A	1.3777 (17)	C13B—H13B	0.9500
C13A—H13A	0.9500	C14B—H14B	0.9500
C14A—H14A	0.9500	C15B—C16B	1.3770 (18)
C15A—C16A	1.3757 (18)	C15B—H15B	0.9500
C15A—H15A	0.9500	C16B—H16B	0.9500
			
N1S—C1S—S1S	178.91 (14)	C11B—N1B—C12B	127.11 (11)
C11A—N1A—C12A	126.84 (11)	C11B—N1B—H1BA	116.4
C11A—N1A—H1AA	116.6	C12B—N1B—H1BA	116.4
C12A—N1A—H1AA	116.6	C15B—N2B—C14B	117.55 (11)
C14A—N2A—C15A	119.63 (11)	C15B—N2B—H2BB	121.2
C14A—N2A—H2AA	120.2	C14B—N2B—H2BB	121.2
C15A—N2A—H2AA	120.2	C2B—C1B—C10B	119.21 (11)
C2A—C1A—C10A	119.38 (12)	C2B—C1B—C11B	124.68 (11)
C2A—C1A—C11A	124.32 (12)	C10B—C1B—C11B	116.11 (11)
C10A—C1A—C11A	116.29 (12)	C1B—C2B—C3B	121.33 (12)
C1A—C2A—C3A	121.01 (12)	C1B—C2B—H2BA	119.3
C1A—C2A—H2AB	119.5	C3B—C2B—H2BA	119.3
C3A—C2A—H2AB	119.5	C4B—C3B—C2B	121.82 (12)
C8A—C3A—C4A	119.61 (13)	C4B—C3B—C8B	119.12 (12)
C8A—C3A—C2A	119.03 (12)	C2B—C3B—C8B	119.06 (12)
C4A—C3A—C2A	121.36 (13)	C5B—C4B—C3B	120.68 (15)
C5A—C4A—C3A	120.27 (15)	C5B—C4B—H4BA	119.7
C5A—C4A—H4AA	119.9	C3B—C4B—H4BA	119.7
C3A—C4A—H4AA	119.9	C4B—C5B—C6B	120.25 (15)
C4A—C5A—C6A	120.28 (15)	C4B—C5B—H5BA	119.9
C4A—C5A—H5AA	119.9	C6B—C5B—H5BA	119.9
C6A—C5A—H5AA	119.9	C7B—C6B—C5B	120.65 (13)
C7A—C6A—C5A	120.78 (13)	C7B—C6B—H6BA	119.7
C7A—C6A—H6AA	119.6	C5B—C6B—H6BA	119.7
C5A—C6A—H6AA	119.6	C6B—C7B—C8B	120.55 (14)
C6A—C7A—C8A	120.49 (15)	C6B—C7B—H7BA	119.7
C6A—C7A—H7AA	119.8	C8B—C7B—H7BA	119.7
C8A—C7A—H7AA	119.8	C9B—C8B—C3B	118.65 (12)
C9A—C8A—C3A	119.19 (12)	C9B—C8B—C7B	122.67 (13)
C9A—C8A—C7A	122.24 (14)	C3B—C8B—C7B	118.68 (13)
C3A—C8A—C7A	118.57 (13)	C10B—C9B—C8B	121.09 (12)
C10A—C9A—C8A	120.63 (13)	C10B—C9B—H9BA	119.5
C10A—C9A—H9AA	119.7	C8B—C9B—H9BA	119.5
C8A—C9A—H9AA	119.7	C9B—C10B—C1B	120.62 (12)
C9A—C10A—C1A	120.75 (13)	C9B—C10B—H10B	119.7
C9A—C10A—H10A	119.6	C1B—C10B—H10B	119.7
C1A—C10A—H10A	119.6	O1B—C11B—N1B	122.34 (11)
O1A—C11A—N1A	122.82 (11)	O1B—C11B—C1B	121.19 (11)
O1A—C11A—C1A	120.67 (12)	N1B—C11B—C1B	116.47 (11)
N1A—C11A—C1A	116.51 (11)	C13B—C12B—N1B	124.48 (11)
N1A—C12A—C16A	117.80 (11)	C13B—C12B—C16B	117.79 (11)
N1A—C12A—C13A	124.03 (12)	N1B—C12B—C16B	117.72 (11)
C16A—C12A—C13A	118.16 (11)	C14B—C13B—C12B	118.76 (12)
C14A—C13A—C12A	118.88 (12)	C14B—C13B—H13B	120.6
C14A—C13A—H13A	120.6	C12B—C13B—H13B	120.6
C12A—C13A—H13A	120.6	N2B—C14B—C13B	123.50 (12)
N2A—C14A—C13A	122.28 (12)	N2B—C14B—H14B	118.3
N2A—C14A—H14A	118.9	C13B—C14B—H14B	118.3
C13A—C14A—H14A	118.9	N2B—C15B—C16B	123.19 (12)
N2A—C15A—C16A	121.34 (12)	N2B—C15B—H15B	118.4
N2A—C15A—H15A	119.3	C16B—C15B—H15B	118.4
C16A—C15A—H15A	119.3	C15B—C16B—C12B	119.21 (12)
C15A—C16A—C12A	119.70 (12)	C15B—C16B—H16B	120.4
C15A—C16A—H16A	120.1	C12B—C16B—H16B	120.4
C12A—C16A—H16A	120.1		
			
C10A—C1A—C2A—C3A	1.0 (2)	C10B—C1B—C2B—C3B	1.06 (19)
C11A—C1A—C2A—C3A	−178.25 (12)	C11B—C1B—C2B—C3B	−178.88 (11)
C1A—C2A—C3A—C8A	0.0 (2)	C1B—C2B—C3B—C4B	−178.85 (13)
C1A—C2A—C3A—C4A	179.46 (13)	C1B—C2B—C3B—C8B	0.88 (19)
C8A—C3A—C4A—C5A	0.4 (2)	C2B—C3B—C4B—C5B	−178.25 (14)
C2A—C3A—C4A—C5A	−179.09 (14)	C8B—C3B—C4B—C5B	2.0 (2)
C3A—C4A—C5A—C6A	0.1 (2)	C3B—C4B—C5B—C6B	0.2 (2)
C4A—C5A—C6A—C7A	0.0 (2)	C4B—C5B—C6B—C7B	−1.5 (2)
C5A—C6A—C7A—C8A	−0.6 (2)	C5B—C6B—C7B—C8B	0.6 (2)
C4A—C3A—C8A—C9A	179.51 (13)	C4B—C3B—C8B—C9B	177.77 (13)
C2A—C3A—C8A—C9A	−0.98 (19)	C2B—C3B—C8B—C9B	−1.97 (18)
C4A—C3A—C8A—C7A	−1.1 (2)	C4B—C3B—C8B—C7B	−2.89 (19)
C2A—C3A—C8A—C7A	178.46 (12)	C2B—C3B—C8B—C7B	177.37 (12)
C6A—C7A—C8A—C9A	−179.41 (13)	C6B—C7B—C8B—C9B	−179.07 (13)
C6A—C7A—C8A—C3A	1.2 (2)	C6B—C7B—C8B—C3B	1.6 (2)
C3A—C8A—C9A—C10A	1.1 (2)	C3B—C8B—C9B—C10B	1.13 (19)
C7A—C8A—C9A—C10A	−178.35 (13)	C7B—C8B—C9B—C10B	−178.18 (13)
C8A—C9A—C10A—C1A	−0.1 (2)	C8B—C9B—C10B—C1B	0.8 (2)
C2A—C1A—C10A—C9A	−0.9 (2)	C2B—C1B—C10B—C9B	−1.93 (19)
C11A—C1A—C10A—C9A	178.39 (12)	C11B—C1B—C10B—C9B	178.01 (12)
C12A—N1A—C11A—O1A	0.2 (2)	C12B—N1B—C11B—O1B	−0.2 (2)
C12A—N1A—C11A—C1A	−179.59 (11)	C12B—N1B—C11B—C1B	179.44 (11)
C2A—C1A—C11A—O1A	174.42 (13)	C2B—C1B—C11B—O1B	178.11 (13)
C10A—C1A—C11A—O1A	−4.83 (18)	C10B—C1B—C11B—O1B	−1.83 (18)
C2A—C1A—C11A—N1A	−5.75 (19)	C2B—C1B—C11B—N1B	−1.58 (18)
C10A—C1A—C11A—N1A	175.00 (11)	C10B—C1B—C11B—N1B	178.48 (11)
C11A—N1A—C12A—C16A	173.68 (12)	C11B—N1B—C12B—C13B	−8.2 (2)
C11A—N1A—C12A—C13A	−5.9 (2)	C11B—N1B—C12B—C16B	172.51 (12)
N1A—C12A—C13A—C14A	−179.35 (12)	N1B—C12B—C13B—C14B	−178.54 (12)
C16A—C12A—C13A—C14A	1.12 (18)	C16B—C12B—C13B—C14B	0.80 (18)
C15A—N2A—C14A—C13A	0.1 (2)	C15B—N2B—C14B—C13B	0.5 (2)
C12A—C13A—C14A—N2A	−0.9 (2)	C12B—C13B—C14B—N2B	−0.9 (2)
C14A—N2A—C15A—C16A	0.4 (2)	C14B—N2B—C15B—C16B	0.0 (2)
N2A—C15A—C16A—C12A	−0.1 (2)	N2B—C15B—C16B—C12B	0.0 (2)
N1A—C12A—C16A—C15A	179.78 (12)	C13B—C12B—C16B—C15B	−0.39 (19)
C13A—C12A—C16A—C15A	−0.65 (19)	N1B—C12B—C16B—C15B	179.00 (12)

### Hydrogen-bond geometry (Å, º)

**Table:**

*D*—H···*A*	*D*—H	H···*A*	*D*···*A*	*D*—H···*A*
N1*A*—H1*AA*···N1*S*	0.88	2.24	3.0576 (17)	154
N1*B*—H1*BA*···S1*S*^i^	0.88	2.69	3.5345 (11)	162
N2*A*—H2*AA*···N2*B*	0.88	1.77	2.6492 (15)	173
N2*B*—H2*BB*···N2*A*	0.88	1.77	2.6492 (15)	177

Symmetry code: (i) *x*, *y*−1, *z*.
